# Genome‐wide expression quantitative trait locus analysis in a recombinant inbred line population for trait dissection in peanut

**DOI:** 10.1111/pbi.13246

**Published:** 2019-09-17

**Authors:** Li Huang, Xia Liu, Manish K. Pandey, Xiaoping Ren, Haiwen Chen, Xiaomeng Xue, Nian Liu, Dongxin Huai, Yuning Chen, Xiaojing Zhou, Huaiyong Luo, Weigang Chen, Yong Lei, Kede Liu, Yingjie Xiao, Rajeev K. Varshney, Boshou Liao, Huifang Jiang

**Affiliations:** ^1^ Key Laboratory of Biology and Genetic Improvement of Oil Crops Ministry of Agriculture Oil Crops Research Institute of the Chinese Academy of Agricultural Sciences Wuhan China; ^2^ National Key Laboratory of Crop Genetic Improvement Huazhong Agricultural University Wuhan China; ^3^ Novogene Bioinformatics Technology Co., Ltd Beijing China; ^4^ Center of Excellence in Genomics and Systems Biology International Crops Research Institute for the Semi‐Arid Tropics (ICRISAT) Hyderabad India

**Keywords:** expression quantitative trait loci (eQTLs), peanut, gene expression variation, RNA‐based sequencing (RNA‐seq), testa colour

## Abstract

The transcriptome connects genome to the gene function and ultimate phenome in biology. So far, transcriptomic approach was not used in peanut for performing trait mapping in bi‐parental populations. In this research, we sequenced the whole transcriptome in immature seeds in a peanut recombinant inbred line (RIL) population and explored thoroughly the landscape of transcriptomic variations and its genetic basis. The comprehensive analysis identified total 49 691 genes in RIL population, of which 92 genes followed a paramutation‐like expression pattern. Expression quantitative trait locus (eQTL) analysis identified 1207 local eQTLs and 15 837 distant eQTLs contributing to the whole‐genome transcriptomic variation in peanut. There were 94 eQTL hot spot regions detected across the genome with the dominance of distant eQTL. By integrating transcriptomic profile and annotation analyses, we unveiled a putative candidate gene and developed a linked marker InDel02 underlying a major QTL responsible for purple testa colour in peanut. Our result provided a first understanding of genetic basis of whole‐genome transcriptomic variation in peanut and illustrates the potential of the transcriptome‐aid approach in dissecting important traits in non‐model plants.

## Introduction

In plant and human genetics, any variation of observable and measurable traits is traceable to DNA sequence mutations. Many studies provided compelling evidence that genetic and epigenetic variations contribute to abundant phenotypic variation in traits via regulating transcript abundance (Albert and Kruglyak, 2015; Chen, 2007; Majewski and Pastinen, 2011). Quantitative trait locus (QTL) mapping or linkage mapping based on segregating populations is a popular and successful approach for identifying the links of DNA sequence variation and phenotypes (Xing and Zhang, 2010). Plethora of literature available based on several studies suggests that the functional mutations may be located in a gene that codes for transcriptional factor or somewhere upstream or downstream of a gene, which allows modulating large amount of gene transcription. More and more empirical evidences are emerging to support the significance of the transcriptomic regulation on ultimate phenotypic traits in plants (Gou *et al*., 2011; Jiao *et al*., 2010; Li *et al*., 2014; Liu *et al*., 2015a,b; Shi *et al*., 2019; Wang *et al*., 2009). In maize, the domestication and genetic improvement were found to be more prevalently associated with transcriptomic and metabolic variations than genomic variations, perhaps due to the fact that the mutation of acid amino changes may bring large side effects on plant survival or adaptation to specific environments (Liu *et al*., 2015a, 2015b).

Propelled by high‐throughput and low‐cost sequencing technology, the gene expression measured using RNA‐based sequencing (RNA‐seq) could be considered for performing QTL mapping for target phenotypic traits in crop plants (Jansen and Nap, 2001). Such analysis will help in exploring the transcriptome variation in segregating populations using expression quantitative trait locus (eQTL) analysis that aims to identify the genomic regions containing DNA sequence variants that regulate the expression level of one or more genes for the target trait (Albert and Kruglyak, 2015; Kliebenstein, 2009; Majewski and Pastinen, 2011). Genome‐wide eQTL mapping was firstly reported in yeast in 2002 (Brem *et al*., 2002) and has been successfully applied for performing genetic or association mapping in plants such as Arabidopsis (DeCook *et al*., 2006; Lowry *et al*., 2013; West *et al*., 2007), rice (Wang *et al*., 2010, 2014), maize (Fu *et al*., 2013; Li *et al*., 2013; Liu *et al*., 2017), tomato (Giovannoni, 2018) and lettuce (Zhang *et al*., 2017). These studies expanded the understanding on landscape of transcriptomic variation within genome, thereby enhancing the understanding of quantitative variations.

Peanut or groundnut (*Arachis hypogaea* L.) is an important and globally cultivated oil seed crop in addition to being a good source of proteins and other micronutrients, such as vitamins, isoflavonoids and phytosterols (Toomer, 2018). In contrast to model plants, peanut has the polyploid genome and self‐mating system, which may raise an open question regarding the general genetic basis of peanut transcriptomic variations. In 2016, the reference sequences of its two diploid ancestors were released ( http://www.peanutbase.com; Bertioli *et al*., 2016). Fuelled by the advancement on sequencing technologies, the draft genomes provided an unprecedented opportunity for the researchers to explore the transcriptomic variation in peanut populations. The understanding of whole‐genome transcriptomic pattern in peanut would be expected to systematically figure out how interactions between genomic and transcriptomic layers can contribute to phenotypic variations. This knowledge would even be beneficial to discover the putative candidate genes for the detected QTLs controlling ultimate phenotypic traits, especially in peanut. Given the fact that peanut has very low seed rate per plant, it seemed unlikely to narrow down QTL regions so far due to unavailability of huge mapping population and sufficient recombination.

In the present study, we sequenced the whole transcriptome of peanut immature seeds after flowering 30 days sampled in a recombinant inbred line (RIL) population derived from crossing Zhonghua 10 (pink testa) and ICG 12625 (purple testa). We explored the landscape of transcriptomic patterns and performed the eQTL analysis to dissect the regulatory network in peanut. Finally, A QTL of testa colour as an example was analysed to illustrate how transcriptomic analyses could help narrow a wide‐spanned QTL to a handle able list of candidate genes, rather than time‐ and labour‐expensive stepwise map‐based cloning strategy. Our result provides a first understanding of genetic basis of whole‐genome transcriptomic variation in peanut and demonstrates its utility for dissecting genes of important traits in non‐model plants.

## Results

### The gene expression variations in peanut immature seed

The immature seed of two parents, Zhonghua 10 and ICG 12625, and 100 RILs were collected 30 days after flowering and sequenced for RNA profiling. A total of 530 Gb clean data and 5.06 billion reads were obtained with 47.7 million reads per RIL on average after aligning to the reference genomes (version G1) of two diploid ancestors, *A. duranensis* V14167 and *A. ipaensis* K30076 ( http://www.peanutbase.com). The proportion of total reads mapped on to two diploid ancestor genomes ranged from 84.5% to 88.6% in the RIL population (Data S1). If the fragments per kilobase per million reads (FPKM) value of one gene was 0 based on the RNA‐seq, we have no idea to confirm whether the gene was not expressed at all or the gene was not detected using RNA‐seq. Thus, we conducted global permutation tests to confirm the threshold value of detectable gene expression and the threshold was 0.047 (false discovery rate, FDR < 0.05). Among uniquely mapped reads, a total of 62 367 genes were found to be significantly expressed in at least one RIL compared to the expected value by chance (FPKM > 0.047). Of all called genes, there were 49 691 genes that expressed in both parents and more than 90% of RILs, which were employed in subsequent analyses.

**Figure 1:**
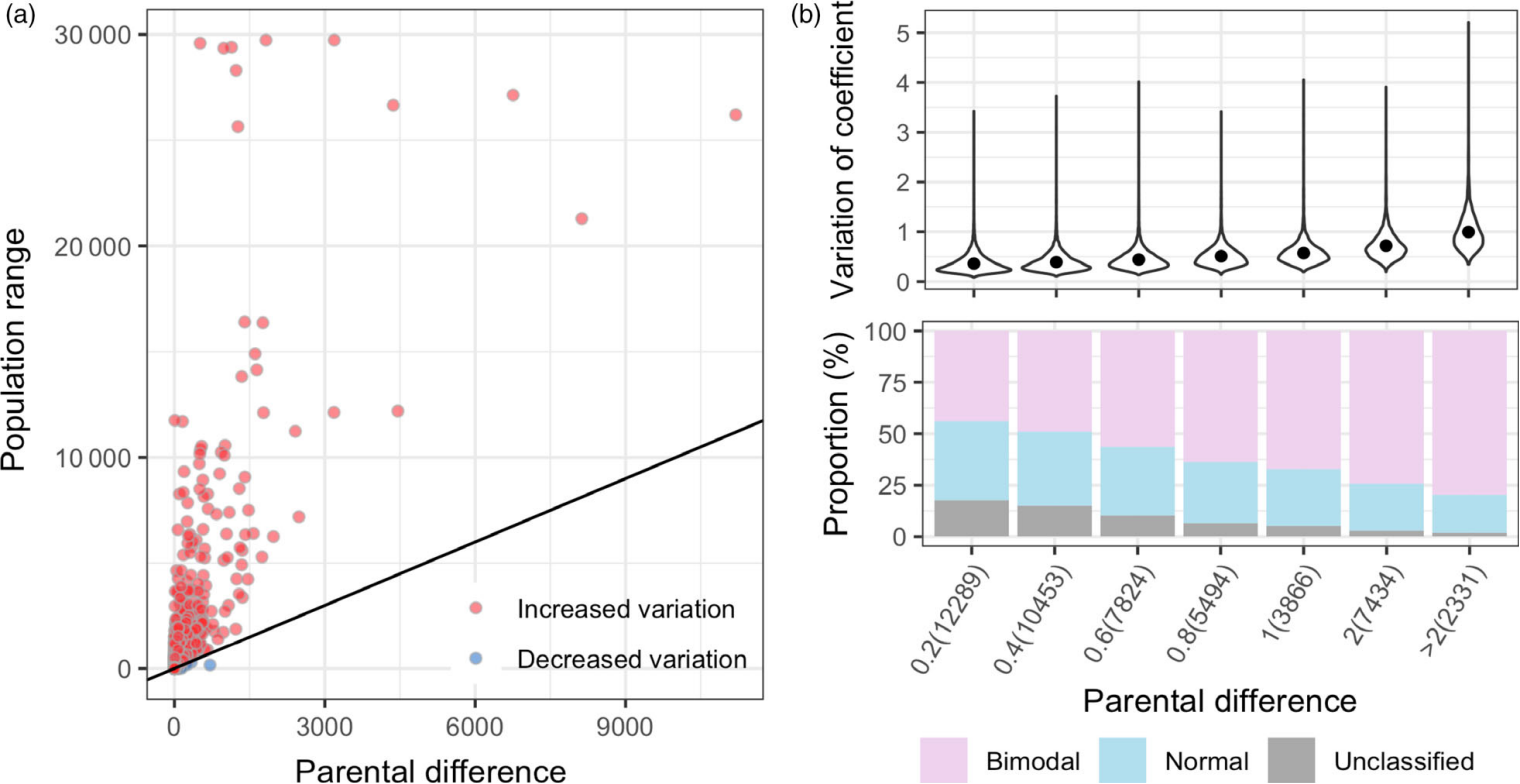
Dynamic population expression variation response to parental difference. (a) The expression variation between parents and population. For each gene, the *x*‐axis of a dot measured the absolute value of expression difference between parents, while the *y*‐axis measured the population range. (b) The relations between population expression variations and distribution with parental differences. The population expression variation was measured by the coefficient of variation (top panel), and the gene expression distributions were categorized as bimodal, normal and unclassified types (bottom panel). In both panels, all genes were grouped by parental differences (*x*‐axis), measured as the absolute value of log2 of expression level in Zhonghua 10 divided by the level in ICG 12625. The numbers in parenthesis show the gene numbers in each category.

Among 49 691 genes, a total of 9765 genes were found differentially expressed (DEGs) with more than twofold changes between parents, Zhonghua 10 and ICG 12625. A total of 3499 DEGs showed higher expression in Zhonghua 10, while 6266 in ICG 12625 (Data S2). In the RIL population, over 99% genes showed much broader variation range in their expression in RIL population as compared to both the parents (Figure 1a), which may be attributed to reinvention of transcriptomic interaction due to the whole‐genome reshuffling during meiosis process. However, we can find that the expression variation in the population (measured as coefficient of variation, CV) strongly depend on the initial parents’ expression difference (Pearson's correlation coefficient, *r *=* *0.58, *P *<* *2.2E−16; Figure 1b), implying the high heritability of expression variations across generations. Three types of distribution patterns for gene expression were identified from the population expression data (Data S2). There were 28 392 (57.1%) genes expressed in a bimodal distribution, while the expression of 15 928 (32.1%) genes exhibited a normal distribution, leaving 5371 (10.8%) genes as unclassified distributions. The phenomenon that the major proportion of genes following bimodal‐expressed patterns probably provided an implicit clue for non‐polygenic feature in the transcriptomic layer. Interestingly, it was found that the proportion of bimodal genes was obviously proportional to the initial expression difference between parents (Figure 1c).

**Figure 2:**
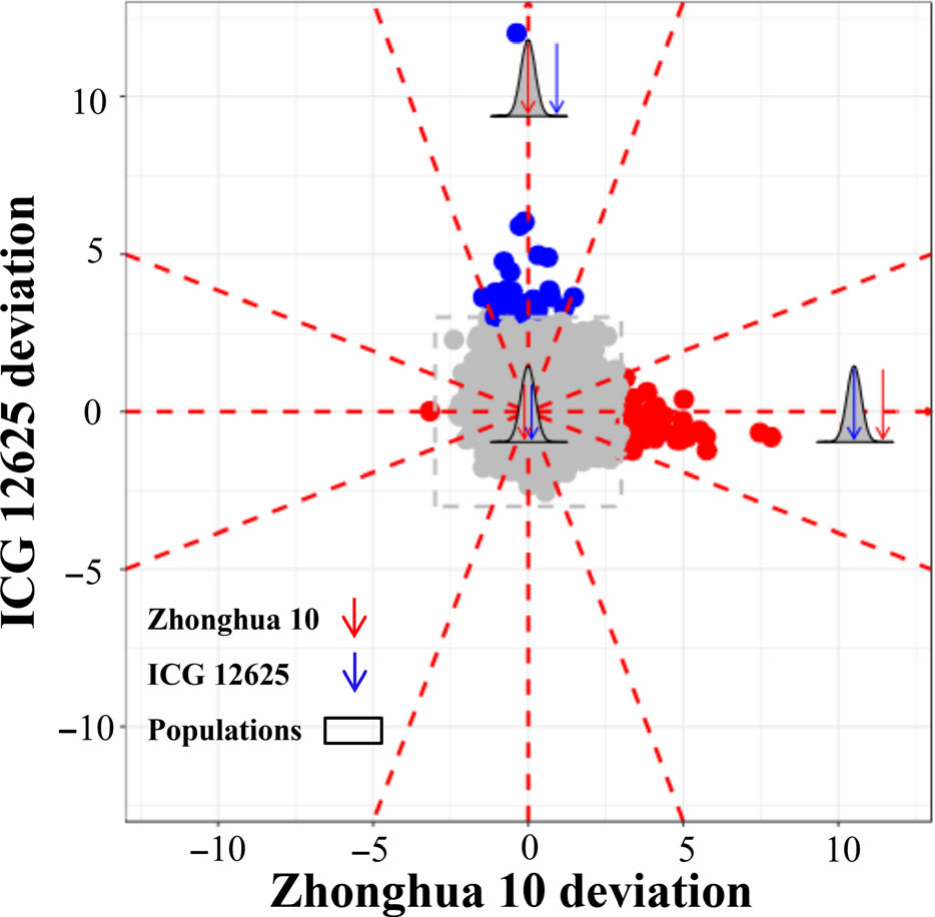
The landscape of gene expression heritable patterns in peanut. Each dot showed the gene expression deviation of two parents to the population mean, measured as the times of the population standard deviation (SD). The genes with more than 3‐SD deviation were regarded as paramutation‐like genes and presented in blue (Zhonghua 10 deviation) or red (ICG 12625 deviation) dots, otherwise as Mendelian‐like genes and presented in grey dots. Totally, 49 691 genes were involved in this analysis.

Paramutation is a genetic variant that apparently violates Mendel's principle of genetic segregation, due to the interaction between paramutable allele and paramutagenic allele in a heterozygote, resulting in changing the phenotype of paramutable allele to that of paramutagenic allele. The genes with paramutation‐like expression indicate that the offspring expression is highly distorted towards one parent. To investigate the inheriting pattern of gene expression, we compared the population mean expression with the initial expression of parents for each gene. It was found that, for the majority of 49 691 genes, the population mean expression showed roughly approximate to the parents’ mean expression (Figure 2). It suggested that the Mendelian principle generally ruled the genetics in the transcriptomic layer, perhaps mediated by the genome‐layer variants. Interestingly, however, we found that the expression of 92 genes in the population apparently departed from the parents’ mean expression, following a paramutation‐like expression pattern. The paramutation‐like genes distorted the population expression mean towards one parent, whereas the other parent extremely expressed with at least three times of standard deviation from the population mean (Figure 2). Within 92 paramutation‐like genes, 50 genes followed a bimodal distribution, and 37 and five genes appeared to be normal and unclassified distribution, respectively, implying there was no significant relevance between the paramutation‐like identity and population distribution (*P *>* *0.05; chi‐squared test). Besides, nearly all detected paramutation‐like genes revealed a uniform expression distortion of population mean towards the low‐expression parent, with one gene exception that expressed distorted towards ICG 12625 as the high‐expression parent (Figure 2).

### RNA‐seq‐based SNP calling and genetic map construction

Based on the reads uniquely mapped to the reference sequences, we totally called 123 039 and 157 248 SNPs from Zhonghua 10 and ICG 12625, respectively, and 51 206–131 142 SNPs from the RILs. By merging SNPs from all lines, a total of 26 300 polymorphic SNPs were obtained due to the low diversity between parents in peanut. Of which, 5768 SNPs were found to be polymorphic between parents and had the missing rate less than 0.2 in RIL population. From 5768 SNPs, a subset of 1285 SNPs were clearly called to be the homozygous genotype for both parents, named as the core SNP set. Considering the genomic complexity of tetra‐polyploid peanut, we used the core SNP set to construct genetic map to avoid the inference bias due to hemi‐SNPs that segregate between homoeologous regions but not actually segregate between different genomes. The majority of core SNPs were allelic transition‐type, nearly twofold of the SNPs with allelic transversion‐type, more details of SNP features are shown in Data S3.

A genetic map was constructed with a total map length of 1911.57 cM and an average map density of 1.47 cM per loci (Figure S1, Table S1). The 20 linkage groups were designated as A01–A10 (A subgenome) and B01–B10 (B subgenome) based on previously reported simple sequence repeats (SSRs; Table S2). Synteny analysis revealed high co‐linearity between genetic map and the physical map (reference genome) of two wild diploid ancestors, albeit a small fraction of inverted segments existed (Figure S2). The linkage groups varied in genetic length that was proportional to the physical length, which accordantly revealed the significantly larger A subgenome than B subgenome (*P *<* *0.05, *t* test; Table S1). The genetic map covered approximately 96% of diploid peanut reference genomes, that is 94.3% for A subgenome and 98.0% for B subgenome (Table S1). Nevertheless, the overall marker density fluctuated on the genetic linkage map, probably due to inherited self‐mating system and a limited population size. The B02 linkage group, for instance, had the smallest flanking interval of 0.99 cM on average, twofold smaller than the B07 linkage group (2.08 cM on average) (Table S1).

### Genome‐wide eQTL analysis in peanut

**Figure 3:**
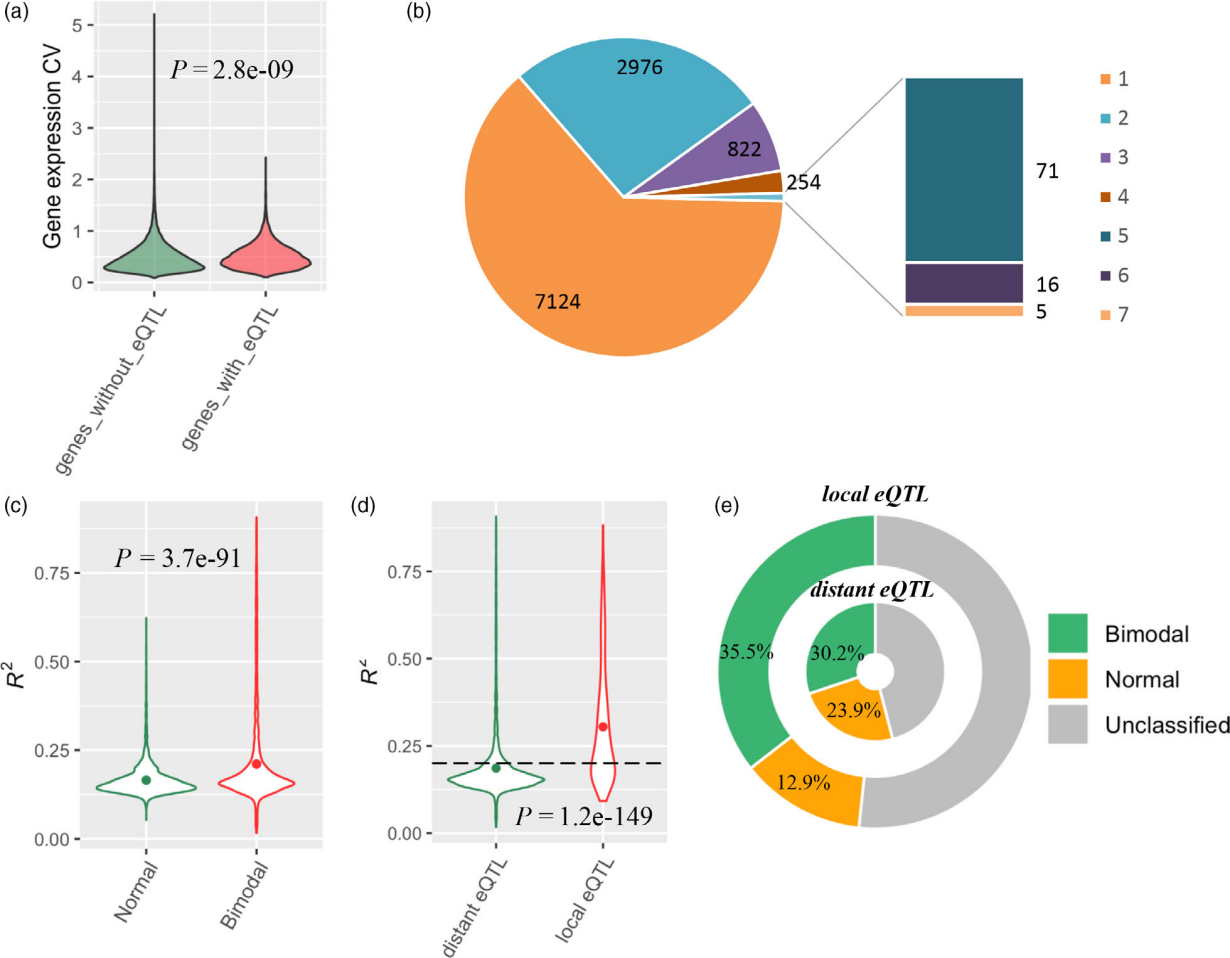
The features of eQTLs. (a) The relationship between eQTL identification and population expression variation. (b) Summary of genes identifying different number of eQTLs. (c) The relationship between gene expression distribution and eQTL‐explained variance. (d) The relationship between explained variance and eQTL type. The dot within violin plot indicates the mean value. The *P* value indicates the significance of difference between groups based on *t* test. The dash line represents 20% of explained gene expression variation for eQTL. (e) The proportions of genes with three distributions regulated by local and distant eQTLs

By treating gene expression as a quantitative trait, a global eQTL analysis was performed for 49 691 genes in RIL population. Totally, 17 044 eQTLs were detected to regulate the expression variation of 11 268 genes in the RIL population (LOD > 4.19; Figure S3). The approximately three quarters of all genes did not detect any eQTL, which may be partly due to the significantly lower population expression variation than genes that detected eQTL (*P *=* *2.8E−09, *t* test; Figure 3a, Data S4). The majority of the genes (7124) were merely controlled by single eQTL, nearly twice more than genes (2976) with two eQTLs. There were ~1% genes (92) that detected more than four eQTLs, among which five genes were found to be affected by seven eQTLs per gene (Figure 3b). The eQTLs for bimodal‐expressed genes (3446) were found to explain 21.1% expression variance on average, significantly higher than the eQTL for normal‐expression genes (*P *<* *0.01, *t* test; Figure 3c). Interestingly, there were 108 eQTLs with more than 70% of the explained variation of gene expression, of which the majority genes (94.4%) followed the bimodal distribution (Data S4).

On the basis of whether an eQTL regulates the gene expression nearby or far away, all eQTLs were designated into 1207 local eQTLs (7%) and 15 837 distant eQTLs (93%) (Data S4). On average, the local eQTLs explained 27.6% of gene expression variation, significantly outweighed distant eQTLs (*P *=* *1.2E−149, *t* test; Figure 3d), despite distant eQTLs seemed to be more prevalent in determining overall transcriptomic variation. Overall, there were 57.33% local eQTLs, which could explain >20% gene expression variation, but only 19.48% distant eQTLs had explained expression variation exceeding 20% (Figure 3d). In local eQTLs, the influenced genes had 35.5% with bimodal distribution and 12.9% with normal distribution. In contrast, for the genes influenced by distant eQTLs, the bimodal proportion decreased to 30.2%, while the normal proportion increased nearly twofold, up to ~24% (Figure 3e). It suggested that local genetic variant might be one important source of genetic basis in the gene expression with non‐polygenic feature.

### The eQTL hot spots play vital roles in transcriptomic variation

**Figure 4:**
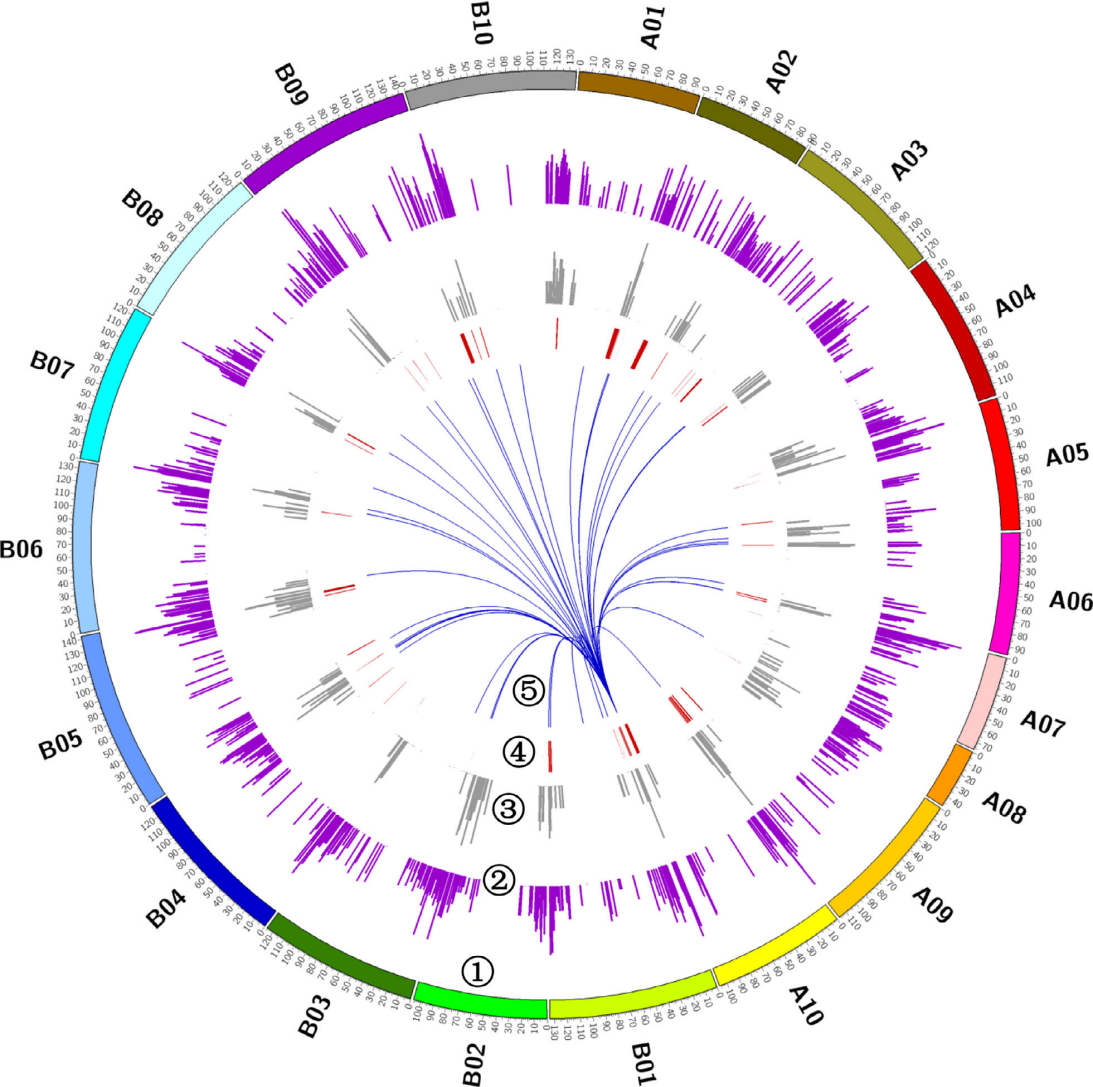
The distribution of eQTL hot spots in the genome. The layers from outer to inner showed the following: ➀ twenty chromosomes of peanut; ➁ the frequency of distant eQTLs along the chromosomes; ➂ the frequency of local eQTLs along the chromosomes; ➃ the regions of eQTL hot spots; and ➄ a case of eQTL hot spot capable to regulate widespread gene expression alteration. This eQTL hot spot fell into the QTL interval on chromosome A10 for purple testa in peanut.

The identified eQTLs dispersed unevenly across the whole genome, ranging from 374 eQTLs on chromosome B08 to 2415 eQTLs on chromosome B10. There were five chromosomes (A07, A09, A10, B09 and B10) carried even over 1000 eQTLs, approximately fivefold higher than expected by chance assuming that the eQTLs were evenly located across the genome (Table S3). To precisely explore the distribution of eQTLs along chromosomes, we searched the identified eQTLs at the 1‐cM sliding windows on each linkage group. The eQTL hot spot was detected at one location in which the number of observed eQTLs exceeded the threshold of 24 eQTLs per cM (FDR = 0.05) based on 1000 permutation tests. We detected a total of 94 eQTL hot spots across the whole genome, ranging from 24 to 1559 eQTLs per hot spot (Figure 4; Table S4). The eQTL hot spot regions totally covered 7% of the peanut genome, but involved 8652 eQTLs that accounted for over a half of the total detected eQTLs across genome. In hot spot regions, the distant eQTLs were significantly enriched as compared to whole‐genome level (Figure 4), up to 97.5%, indicating a hypothesis that the eQTL hot spot may function in regulating long‐range gene expression. Gene ontology (GO) analysis was performed for 821 genes on the 9–15 cM interval of chromosome B10, which was an eQTL hot spot identifying most eQTLs. It found that these genes mainly participated in metabolic process, such as macromolecule (GO:0043170, 211 genes), cellular macromolecule (GO:0044260, 191 genes), nitrogen compound (GO:0006807, 177 genes) and protein metabolic process (GO:0019538, 130 genes; Data S5). Among 821 genes, a set of 576 genes had gene annotation, of which 14 genes were transcription factors, such as bHLH, MYB, GLABRA.

### Discovery of candidate genes for purple testa in peanut using transcriptome‐based mapping approach

**Figure 5:**
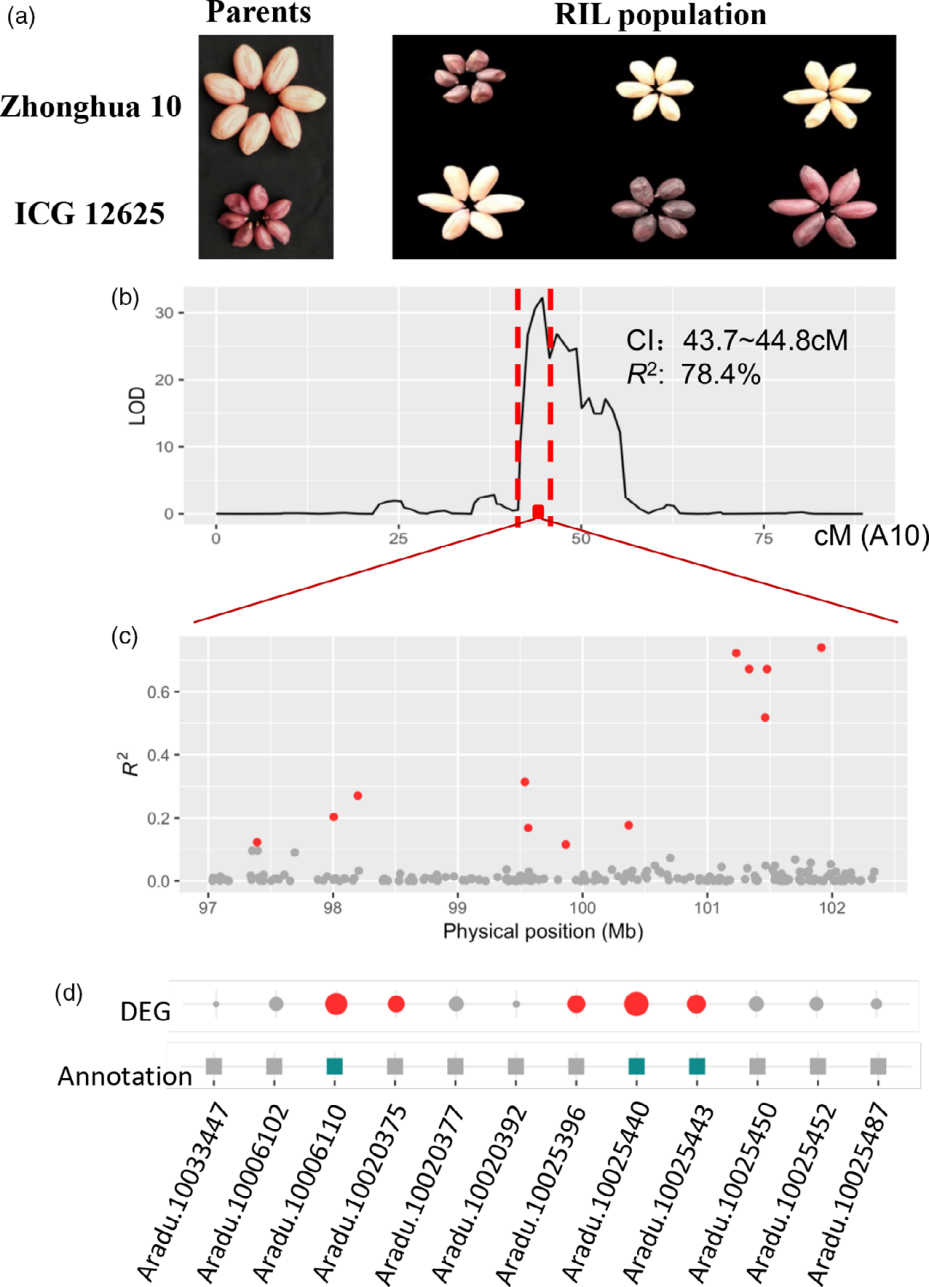
Integrating transcriptomic data empowered rapid gene exploration for purple testa. (a) Phenotype of testa colour in peanut seed. The left panel indicates the Zhonghua 10 (pink) and ICG 12625 (purple), while right panel shows the colour variability in the population. (b) Identification of a major QTL underlying testa colour on chromosome A10. The red rectangle indicates the 99% support interval of the QTL, while the red dash lines mean the closest markers flanking the QTL interval in the map. (c) The relationship between gene expression and testa colour in the population. A total of 196 genes were tested at the candidate region (97.0~102.3 Mb). The genes with *P *<* *0.001 were highlighted as red dots. (d) Integrative analyses help determine putative genes responsible for purple colour. The top layer indicates the extent of expression difference (DE) between parents. The circle size is proportional to the DE level, while the red filled ones mean the significantly different expression based on *t* test (*P *<* *0.01). The middle layer indicates whether the gene expression can be regulated itself, as local eQTL and filled in blue, otherwise in grey. The bottom layer indicates whether the gene may be involved in anthocyanidin biological synthesis pathway according to the peanut and *Arabidopsis* annotation, as filled in green, otherwise in gray.

Exploring the functional genes for target traits is the long‐term and ultimate goal for molecular biologist in order to improve genetics of crops for feeding human population. Despite the success of map‐based cloning strategy proved in rice, it is still a huge challenge in peanut due to seed rate (less number of seeds per plant) problem. Here, we proposed a transcriptome‐based approach to help efficiently determine the putative genes for trait with interests. In the present study, we tempted to use peanut testa colour, a trait with high market value, as an example to illustrate it. The seed testa colour differed between two parents and segregated in the RIL population (Figure 5a). Of the 100 RILs, 52 lines carried light coloured testa and 48 lines had dark coloured testa, which followed the expected segregation ratio (1 : 1, χ^2^ = 0.020, *P *=* *0.887), implying that the colour of seed testa may be caused by the variant of a single locus of the gene. The whole‐genome QTL scanning only detected one QTL (LOD = 32.2) at 43.7–44.8 cM on chromosome A10, explaining 78.5% of phenotypic variance for testa colour (Figure 5b). The closet markers flanking this QTL delimited a bit larger interval (41.7–45.7 cM), equivalent to 84.6–101.6 Mb on chromosome A10. In order to fine‐map this QTL, a residual heterozygous line was used to obtain near‐isogenic lines (NILs) with pink and purple testa colour, respectively. Based on the reference genome sequence, 223 SSR markers were developed at the region of 84.0–103.1 Mb on chromosome A10 and amplified in the parental lines and the NILs with pink and purple testa. The polymorphism analysis of the primers enabled to narrow the QTL interval into a 97 001 012–102 338 287 bp region (Table S5), including 196 genes based on the reference genomes. In order to further explore putative genes, the population‐based transcriptomic data provide us an alternative to traditional approach using large populations. From these 196 genes, the majority of the genes showed weak correlations between gene expression and testa colour in the population, only 12 genes reached the significant correlations with testa colour (*P *<* *0.001; Figure 5c; Table S6). For 12 genes, a two‐step procedure was applied to determine the putative gene. First, DE analysis revealed five genes expressed significantly different between parents (*P *<* *0.01; Table S6). Second, three genes were predicted to be involved in anthocyanidin biological synthesis pathway according to annotation information of *A*. *duranensis* V14167 (Figure 5d; Table S6). Based on these results, three genes (*Aradu.10006110, Aradu.10025440* and *Aradu.10025443*) were predicted to involve in anthocyanidin biological synthesis and present purple testa colours in peanut. Meanwhile, the homology gene of *Aradu.10025443* in tetraploid genome, *Arahy.J3K16K*, and a closely linked SNP marker, pTesta1089, were reported controlling the purple testa in peanut in previous study (Zhao *et al*., 2019). Thus, the present study successfully demonstrated the potential of transcriptome‐based genetic mapping approach in discovery of candidate genes for purple testa in peanut.

**Figure 6:**
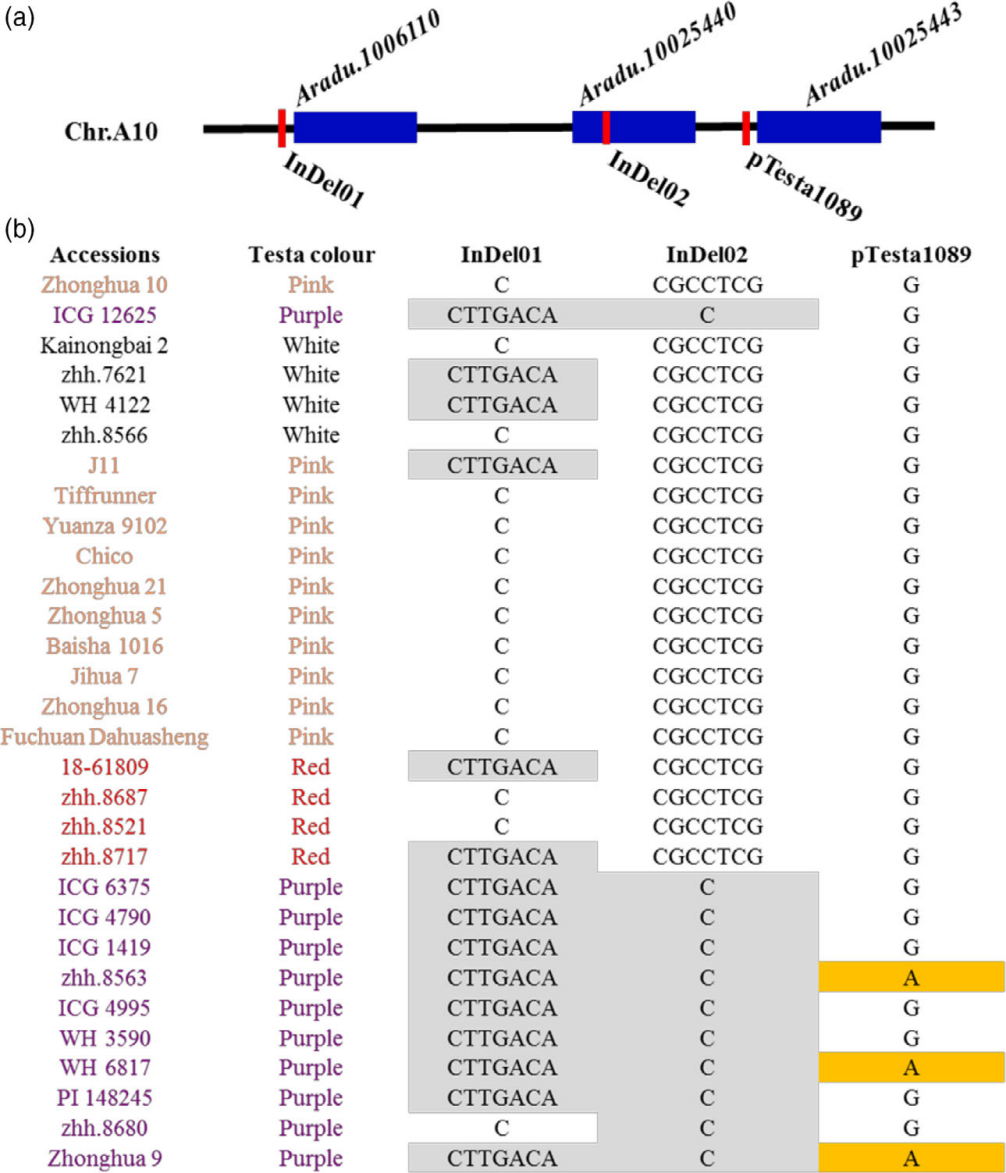
Variations of three markers in parents and other germplasms. (a) The position of three genes and three linked markers on the chromosome A10. The dark blue rectangles represented the gene, and the red thick lines represented the linked markers. (b) The variations of three markers in parents (Zhonghua 10 and ICG 12625) and other germplasms with white, pink, red and purple testa colour. In the first and second lines, the font colour of black, orange, red and purple represented the germplasms with white, pink, red and purple testa colour. The genotypes with grey filling in InDel01 and InDel02 markers represented the same genotype as the purple testa colour parent ICG 12625 in our study. The genotypes with yellow filling in pTesta1089 marker represented the same genotype as the purple testa colour parent Zhonghua 9 in Zhao's previous study.

To identify the DNA sequence variation of candidate genes, the whole‐genome resequencing data (30 Gb) were generated for parents and sequence analysis identified 562 818 genome‐wide SNP/InDel variations. There was an InDel variation between Zhonghua 10 and ICG 12625 for *Aradu.10006110* (C/CTTGACA) and *Aradu.10025440* (CGCCTCG/C), respectively. Based on these variations, two InDel markers located in two genes, InDel01 and InDel02, were designed and used for genotyping parents together with 30 germplasms with different testa colour (Table S7). The amplification in different accessions (Figure 6) indicated that InDel02 marker had highest detection accuracy of purple testa in different peanut accessions. However, we found that the parents had no difference in the locus of pTesta1089 marker and several purple testa accessions had the same genotype as pink, red and white testa accessions (Figure 6). These results indicated that, besides *Arahy.J3K16K*, there may be another gene(s) controlling purple testa in peanut, probably attributed to *Aradu.10025440*.

## Discussion

Recent advances in sequencing technologies made available low‐cost and faster data generation, which accelerated deployment of sequencing‐based applications more frequent in different crop plants for trait mapping and molecular breeding (Pandey *et al*., 2016; Varshney *et al*., 2019). Majority of the earlier sequencing‐based studies were performed by sequencing DNA from segregating genetic populations; however, the RNA sequencing‐based genetic mapping has not yet been used in peanut. In this research, we successfully performed RNA‐seq experiment‐based genetic mapping using a RIL population to explore the landscape of transcriptomic variation on the key stage of kernel development in peanut and discovered genes controlling purple testa colour.

### The genetic basis of the whole‐genome transcriptomic variation in peanut

The genomic variation influences the phenotypic diversity mostly mediated by transcriptomic and metabolic regulations. The present study exhibited tremendous variations in a segregation population for gene expression, which is much higher than the traditional agronomic variation. We found roughly two‐third of the gene expressions following bimodal distribution, indicating the majority of gene expressions to be controlled by single or several large‐effect genes, as qualitative feature. Nearly one quarter of genes followed normal distribution, suggesting the polygenic features also complemented the global genetic basis of transcriptomic variations in peanut. The simple genetic basis of transcriptomic and metabolic layers of variations was previously reported in maize (Liu *et al*., 2017; Wen *et al*., 2015) and Arabidopsis (Wu *et al*., 2016), similar to the findings of present study as tetraploid species. The transcriptome and metabolome are the bridging layers between genome and phenome; therefore, finding association of the transcriptome, being more closer to phenome, becomes more precise reasonable. It would be expected that several large‐effect genes cause the transcriptomic variation. The non‐Mendelian inheritance is an interesting question in genetics, but only few cloned genes proved to act in non‐Mendelian pattern. In maize, the different alleles of *B1* locus made the uniform purple stalk in an F_2_ segregation population, which has been reported to work via epigenetic interaction in the post‐transcriptional layer, denoted as paramutation (Eichten *et al*., 2011). The RNA‐seq in parents and segregating population is a good approach to explore the global overview of non‐Mendelian inheritance or paramutation pattern, which were successfully deployed in maize (Li *et al*., 2013) and tomato (Shivaprasad *et al*., 2012). We presented a pilot study of RNA‐seq experiment on an advanced RIL population in tetraploid peanut and successfully identified 92 paramutation‐like genes, which showed a specific pattern in the RILs being more towards the lower parent. This pattern was previously observed in a maize study, in which the majority of paramutation‐like genes (124/145) were expressed in the population towards the low‐expression parent (Li *et al*., 2013). It was speculated that most examples of paramutation involve that a paramutagenic allele was expressed at lower levels than the paramutable allele.

QTL mapping is a popular and effective tool to dissect quantitative traits in segregating populations, which can be intuitively applied in gene expression as eQTL mapping. In allopolyploid species, it is difficult to differentiate more abundant inter‐homoeologous polymorphisms that are not real single nucleotide polymorphisms, due to the existence of homoeologous sequences (Chen *et al*., 2013; Trick *et al*., 2009). In the present study, we only used core SNPs that were identified from uniquely mapped reads to construct the genetic map. This strategy makes it precise to construct genetic map and perform QTL mapping. Although the homoeologous genes had the same conserved domain, there was still large genomic sequence variation between homoeologous genes. For example, the candidate gene *Aradu.1025440* and its homoeologous gene *Araip.10031835* had 121 genomic sequence variations (99 SNPs and 22 InDels), among which there were 14 (12 SNPs and two InDels) and 107 (87 SNPs and 20 InDels) sequence variations in exon and intron regions, respectively (Figure S4). We identified 17 044 eQTLs for 11 268 expressed genes. The bimodal‐expressed genes apparently had more large‐effect eQTLs than the normal‐expressed genes, which verified the conclusion that genes with bimodal distribution may be caused by simpler genetic base. For all eQTLs, there were 15 837 distant eQTLs that acted in trans, which were 13‐fold more than local eQTLs that acted in cis. Despite the high proportion of distant eQTL, we can see that local eQTL contributes significantly higher effect to transcriptomic variation than distant eQTL. It is reasonable that gene expression follows the polygenic basis, as a quantitative trait. The majority of minor‐effect QTL for gene expression was found to be distantly regulating factors (distant eQTL), which may be biologically efficient that only finite genes are needed to contribute infinite (expression) phenotypic consequences via long‐distance interaction such as transcriptional factor, enhancer or silencer. Similarly, the phenomena of much more distant eQTL than local eQTL have been reported in the previous studies in rice, maize and other species (Liu *et al*., 2017; Wang *et al*., 2010; Zhang *et al*., 2017). Although a huge number of eQTLs were detected in present study, they showed strong physical clustering leading to identification of 94 hot spot regions with eQTLs more than expected by chance. There was also apparent enrichment of distant eQTLs on the hot spot regions compared to the non‐hot spot regions. Taking together, the whole‐genome transcriptomic variation may be controlled by a limited number of genomic regions mediated by high volume of distant eQTLs, which should be biologically economical way to manipulate accurate regulation process (Kliebenstein, 2009; Li *et al*., 2013; Liu *et al*., 2017). Because of limited mapping resolution on bi‐parental population, our eQTL analysis could not detect the causal genes underlying gene expressions. Nevertheless, the present results would provide first understanding of the genetic controls of transcriptomic landscape in peanut. The more comprehensive and high‐resolution exploration of transcriptomic variation in peanut would be expected in more diverse populations and multiple tissues.

### The omics‐based approach benefits efficiently dissecting trait variation

Identification of the genes controlling phenotypic variation plays important role in understanding genetic basis of key traits. The routine QTL mapping and map‐based cloning are prevalent and successful to isolate the underlying genes in model plant species, such as *Arabidopsis* and rice (Gou *et al*., 2011; Jiao *et al*., 2010; Li *et al*., 2014; Wang *et al*., 2009; Xing and Zhang, 2010). However, it is an enormous challenge in peanut, which is hard to produce sufficient recombinations in a small‐size NIL population due to low fecundity. We demonstrated how an integrated approach of genomic and transcriptomic data could provide an opportunity to pinpoint the putative gene responsible for trait of interest, using the seed testa colour as an example. In present study, we detected a major QTL of testa colour in a RIL population, where the segregation of phenotype implied a single‐gene genetic basis for testa colour. The closest flanking marker surrounding the QTL of testa colour enabled to delimit a 17‐Mb region responsible to this QTL on chromosome 10. According to residual heterozygous lines at this QTL, we constructed four different NIL populations with ~600 lines per population and developed 226 new SSR markers dispersed within the 17‐Mb QTL region. The fine‐mapping approach enabled to narrow down the QTL into a 5.2‐Mb region, which still contained 196 genes. It would be impossible to obtain sufficient recombination at the QTL region from thousands of NIL lines in peanut, which may be the routine in map‐based cloning in rice (Li *et al*., 2014; Xue *et al*., 2008). However, like many findings in maize, the gene expression alteration may be dominant in regulating trait variations, especially for traits involved in plant domestication (Liu *et al*., 2015a,b); thus, the deployment of omics data may be a proven tool to efficiently discover candidate genes for metabolic traits (Wen *et al*., 2015). In the present study, we found that the expression differences between parents were inheritable and huge amounts of eQTLs had been identified for expression variation on single gene. Among the 196 genes, by integrating the transcriptomic information for parents and RIL population, correlation between testa colour and gene expression, and gene annotation, we identified that three genes may be strong responsible genes for the testa colour in peanut. Meanwhile, 235 eQTLs that regulated expression level of 201 genes overlapped with the major QTLs for testa colour. Among these three genes, one gene has been reported controlling purple testa colour in peanut in the previous study (Zhao *et al*., 2019), which indicated that transcription analysis of population could effectively and rapidly help identify candidate genes for interested traits. In our study, we tried to identify the DNA sequence variation of purple testa and develop two InDel markers based on resequencing data of parents. In Zhao's study, marker pTesta1089 that linked with purple testa had sequence variation (G/A) between pink testa and purple testa in peanut. However, this marker had no sequence variation between Zhonghua 10 and ICG 12625 in our study, and had the same genotype in several purple testa accessions as pink testa accessions (Figure 6). These results indicated that there may be additional genes controlling purple testa in peanut. Meanwhile, the marker InDel02 developed from gene *Aradu.10025440* sequences had precise identification in accessions with different testa colours. We found that if these six bases (GCCTCG) were absent on the locus of InDel02 marker in one accession, the testa colour of this accession was purple. It indicated that gene *Aradu.10025440* may be a novel gene controlling purple testa in peanut. Furthermore, the function of identified candidate gene needs to be further verified by molecular biological experiments, CRISPR‐Cas9 experiment and RNAi experiment, which were undergoing. Our results nevertheless illuminated a workable solution, in an era of big data, to identify candidate genes based on primarily QTL analysis in peanut, especially for the species without high seed fecundity such as trees.

## Experimental procedures

### Plant materials and sequencing

A peanut RIL population was developed by crossing Zhonghua 10 and ICG 12625, followed by successive selfing for seven generations (Huang *et al*., 2016). The female parent, Zhonghua 10 (*A. hypogaea* var. *vulgaris*), is a cultivar with pink seed coat developed by Oil Crops Research Institute of the Chinese Academy of Agricultural Sciences (OCRI‐CAAS), Wuhan, China, in 2004. The paternal parent, ICG 12625 (PI497597, *A. hypogaea* var. *aequatoriana*), is a germplasm with dark purple seed coat introduced from International Crop Research Institute for the Semi‐Arid Tropics (ICRISAT), Hyderabad, India. The parental lines and RIL population were planted in one‐row plots in an incomplete randomized block design in experimental field in 2014 in OCRI‐CAAS, Wuhan, China.

Five immature seeds from three plants of the two parental lines and 100 lines randomly selected in the RIL population were collected in 30DAF. The seeds (including seed testa) were immediately frozen in liquid nitrogen for RNA extraction. The immature seeds of the parental lines were obtained in three biological replications, and the immature seeds of the RIL population obtained in two biological replications were bulked. Total RNA was extracted using TRIzol Reagent (TaKaRa, Inc., Dalian, China) according to its protocol. RNA degradation and contamination were monitored on 1% agarose gels. RNA quality and purity were checked by Agilent 2100 and NanoDrop. The construction of cDNA libraries was performed from RNA samples for Illumina paired‐end (PE) sequencing following the Illumina protocol. Subsequently, the library preparations were sequenced on an Illumina HiSeq 2000 platform (Illumina, San Diego, CA) and paired‐end reads (2 × 100 bp) were generated at Novogene Bioinformatics Technology Co., Ltd (Beijing, China).

### Reads mapping and SNP calling

After removing low‐quality reads and reads containing adapter or ploy‐N, the remaining paired‐end clean reads were aligned to the reference genomes (version G1) of two diploid ancestors *A. duranensis* V14167 and *A. ipaensis* K30076 ( http://www.peanutbase.com) using TopHat v2.0.12 (Trapnell *et al*., 2009). The SNP calling was performed using SAMtools (Li *et al*., 2009). The reliability of expression data was evaluated by the Pearson correlation coefficients between three biological replications in two parental lines. The reliability of SNP calling was quantified by comparing genotypic consistency among three replicated samples for two parents, respectively. A filtered SNP set was obtained by employing following criterions: (i) any called SNP genotype should be covered by more than two reads (depth ≥2); (ii) the SNP must be polymorphic between parents and within RIL populations; (iii) the minor allele frequency is beyond 0.2 to exclude extremely distorted segregation SNPs; and (iv) the rate of missing and heterozygosity in RIL population should both be less than 0.2, respectively. As other tetraploid species, the peanut SNP calling may have a high probability of identifying hemi‐SNPs that segregate between homoeologous regions but not actually segregate between different genomes (Chen *et al*., 2013). To avoid genotyping bias, we selected a core SNP set that clearly segregates between two parents as two homozygous genotypes in the following analyses.

### Gene expression analysis in RIL population

The Cufflinks v2.1.1 Reference Annotation Based Transcript (RABT) assembly method was used to identify both known and novel genes from TopHat alignment results (Trapnell *et al*., 2010). The names of novel genes had prefix with ‘Novel’. Based on the alignment to the reference genomes of two diploid ancestors ( http://www.peanutbase.com), the FPKM for each gene were calculated using HTSeq v0.6.1 (Anders *et al*., 2015), as the qualification of gene expression. Given the systematic bias due to short‐reads alignment to the tetraploid genome, the FPKM value for each gene was compared to the null distribution of gene expression, obtained by randomly selecting 1 000 000 non‐genic fragments with 1000‐bp length from the RNA‐seq data, estimating the FPKM value and repeating the process 1000 times. The gene expressed significantly higher than the expected by chance if the observed FPKM of gene expression exceeded the 95th percentile of the null distribution (FDR < 0.05). The set of genes that significantly expressed in both parents and more than 90% RIL lines were used in the following analyses.

To quantify expressive variations between parents, we calculated the differential expression for each gene as the absolute value of log2 on the ratio of FPKM between Zhonghua 10 and ICG 12625. The distribution of gene expression in the RIL lines was classified into three categories following the procedures: (i) bimodal distribution, if the threshold of BI value was >1.0 and *P *>* *0.001 using the package R/BiSEp; (ii) normal distribution, if not significantly deviated from a standard normal distribution using the Shapiro–Wilk test (*P *>* *0.01) using the R function ‘shapiro.test’; and (iii) unclassified distribution if it did not follow neither normal distribution nor bimodal distribution. The relationship between coefficients of variation of gene expression in the RIL lines and DE in parents was assessed by Pearson's correlation analysis in R ( http://www.R-project.org). For one gene, if one parent was within the gene expression distribution (two standard deviation from the population mean) of the RILs but the other parent had an expression level at least three standard deviations from the population mean, the gene was considered to be paramutation‐like expression (Li *et al*., 2013).

### Genetic linkage map construction

The core SNP set uniquely mapped to the genome was employed to construct the genetic linkage map, aiming to reduce genotyping bias due to hemi‐SNP between homoeologous regions between subgenomes. Given the core SNP data only covered the genetic variants within genic regions, we selected another set of 306 SSR markers with more frequently coverage on intergenic regions to improve the genetic map. All SSR markers had a uniform distribution on the previously published genetic map (Huang *et al*., 2016) and the reference genomes of two diploid ancestors ( http://www.peanutbase.com) based on the BLAST analysis. The genetic map was constructed using JoinMap 3.0 (Van and Voorrips, 2001) with minimum LOD of 4.0. The genetic distance was generated by Kosambi map function (Kosambi, 1944). The linkage groups were designated as A01~A10 for A subgenome and B01~B10 for B subgenome. The graphic representation of genetic map was generated in R ( http://www.R-project.org). The comparison maps of the genetic position with the physical position for the loci in A and B subgenomes were shown using ‘circlize’ software package in R ( http://www.R-project.org).

### QTL analysis for gene expression and testa colour

To explore the genetic determinants of gene expression and testa colour in peanut, we performed QTL analysis on the variation of gene expression and testa colour in the RIL population. Based on the high‐density genetic linkage map, the composite interval mapping (CIM; Zeng, 1994) was implemented in the software QTL cartographer (Basten *et al*., 2004) for QTL performing analysis, with the siding window size of 30‐cM and walking speed of 1‐cM. For the phenotype of testa colour, an empirical LOD threshold value of 3.0 was used to identify a QTL controlling testa colour. For the gene expression, the permutation tests based on 100 randomly selected genes were performed. In any selected gene, the reshuffled expression data across RILs were used to perform QTL analysis and the largest LOD value was recorded. The 99th percentile of recorded LOD values based on 1000 permutation was declared as the LOD threshold for this gene at the FDR < 0.01. The average of LOD thresholds across 100 random genes was treated as the global LOD threshold (LOD ≥ 4.19) to declare a QTL controlling gene expression, hereafter as eQTL. If the peaks of two adjacent QTLs were in less than 5 cM for the same trait, these two eQTLs were combined and regarded as a single eQTL. The 2‐LOD drop interval from the peak was regarded as the confident interval of QTL at *P *<* *0.01. If the interval of an eQTL colocalized with its influenced gene, the eQTL was considered as a local eQTL, otherwise distant eQTL.

### Identification of eQTL hot spots

To evaluate the distribution of eQTLs across the genome, we summarized the number of eQTLs located within a sliding window of 1‐cM along the chromosome. A permutation test was used to assess whether the number of eQTLs identified in specific locations was significantly more than the expected by chance, assuming the eQTLs uniformly distributed across genome. In each permutation, the total eQTLs were randomly relocated onto the 1‐cM windows across the genome and the largest number of eQTLs per window was recorded. The process was repeated 1000 times, and the 99^th^ percentile of 1000 recorded values was regarded as a threshold declaring a genomic location significantly enriched eQTLs relative to expected by chance (FDR < 0.01), hereafter as eQTL hot spot. The distribution of eQTL hot spots in the genome was generated by ‘circlize’ software package in R ( http://www.R-project.org).

### Variations of linked markers in germplasms

To identify the sequence variations of candidate genes, genome resequencing was performed for the parents using Illumina HiSeq 2500. The DNA was isolated using CTAB method (Huang *et al*., 2016), and DNA‐seq libraries were generated using the TruSeq Nano DNA HT Sample Preparation Kit (Illumina, Novogene Bioinformatics Technology Co., Ltd, Beijing, China). Each parent obtained genome sequencing data about 30 Gb data. After quality control, sequence data were aligned to the reference genomes of two diploid ancestors ( http://www.peanutbase.com) using Burrows–Wheeler Aligner (Li and Durbin, 2009) and repetitive sequences were removed using SAMtools (Li *et al*., 2009). The variation detection including SNPs and InDels between parents was performed with HaplotypeCaller in GATK (McKenna *et al*., 2010). The linked markers were developed based on the sequence variation near or in the candidate genes, and PCR amplification was performed in different germplasms including four white testa colour, 10 pink testa colour, four red testa colour and 10 purple testa colour.

## Conflict of interest

The authors declare no conflict of interest.

## Authors’ contributions

L.H. and H.J. designed the study. X.R. planted the mapping population. X.R., Y.C., X.Z. and W.C. collected immature seeds in the population and extracted the RNA. L.H., X.L., H.C. and X.X. analysed the data. L.H. and H.J. wrote the manuscript. M.K.P., N.L., D.H., H.L., K.L., Y.L., Y.X., R.K.V. and B.L. revised the manuscript. All of the authors read and approved the final manuscript.

## Supporting information


**Figure S1** Genetic map of the RILs derived from Zhonghua 10 and ICG 12625 in peanut.
**Figure S2** Comparison of the genetic position with the physical position for the loci in the genetic map in peanut.
**Figure S3** The overview of eQTLs responsible for whole‐genome gene expression.
**Figure S4** Gene sequences comparing between the candidate gene *Aradu.1025440* and its homoeologous gene *Araip.10031835*.

**Table S1** Statistics of genetic linkage map based on RNA‐seq
**Table S2** The information of the loci in the genetic map in the RILs
**Table S3** Statistics of the eQTLs across the whole genome in peanut
**Table S4** The information of 94 eQTL hotspots across the whole genome in peanut
**Table S5** The information of 223 SSR markers developed in the regions of the major QTL for testa color
**Table S6** The information of 12 genes with significant correlation to the testa color in the RILs in the major QTL (*P *<* *0.001)
**Table S7** The primer sequences of marker InDel01 and InDel02

**Data S1** The mapping information of RIL population based on RNA sequencing.
**Data S2** The summary of gene expression for 49 691 genes in parents and RIL population.
**Data S3** The details of 1285 homozygous SNP detected in present study.
**Data S4** The details of eQTL information detected in present study.
**Data S5** GO analysis of eQTL hotspot on the 9–15 cM interval of chromosome B10.
