# Supplementary material for: Genome‐wide expression quantitative trait locus analysis in a recombinant inbred line population for trait dissection in peanut
Source: Plant Biotechnol J. 2019 Sep 17;18(3):779–90. doi: 10.1111/pbi.13246 (PMC7004917; doi:10.1111/pbi.13246)
Supplement: Supplementary file 1 — Figure S1 Genetic map of the RILs derived from Zhonghua 10 and ICG 12625 in peanut. Figure S2 Comparison of the genetic position with the physical position for the loci in the genetic map in peanut. Figure S3 The overview of eQTLs responsible for whole‐genome gene expression. Figure S4 Gene sequences comparing between the candidate gene Aradu.1025440 and its homoeologous gene Araip.10031835. [file PBI-18-779-s002.docx]

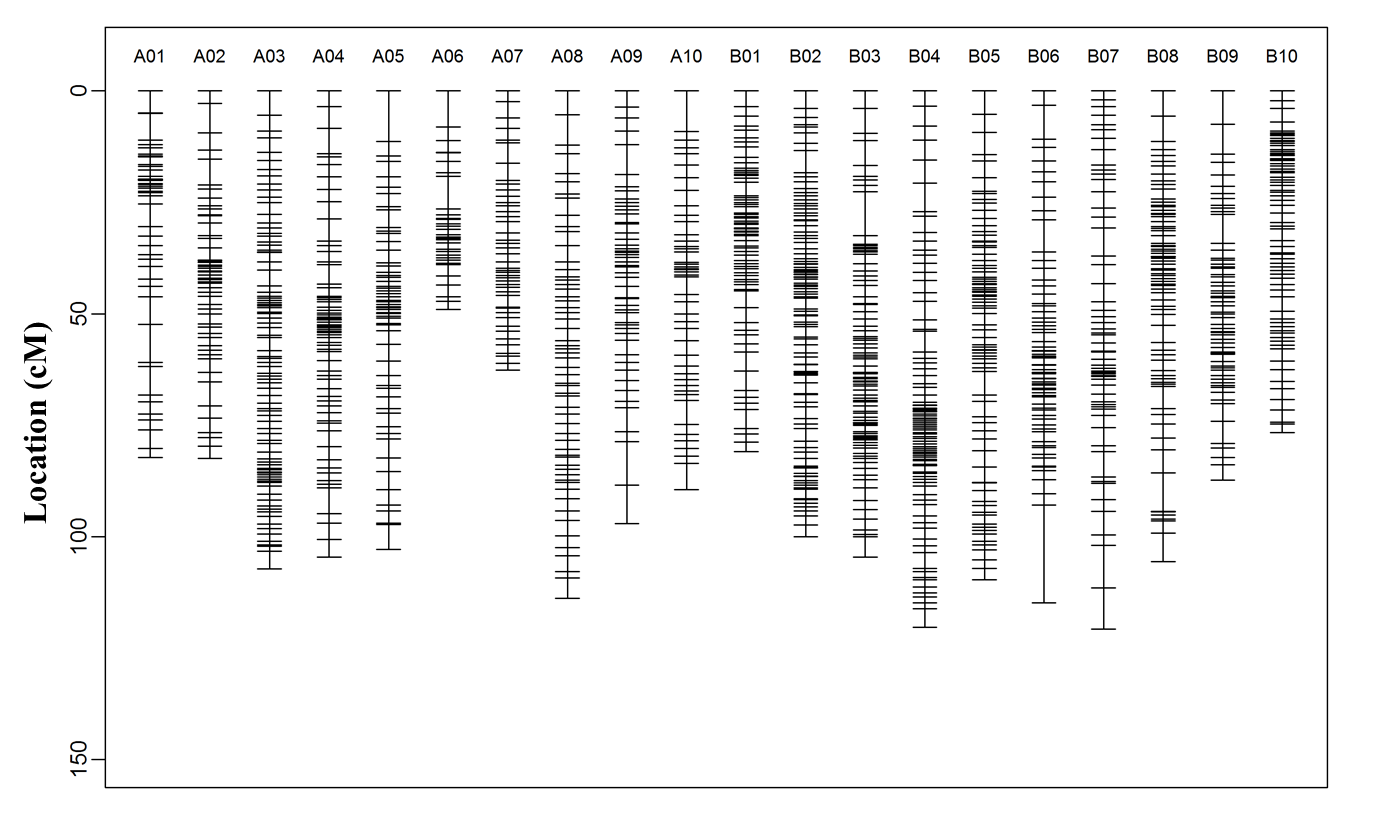


**Figure S1 Genetic map of the RILs derived from Zhonghua 10 and ICG 12625 in peanut.**


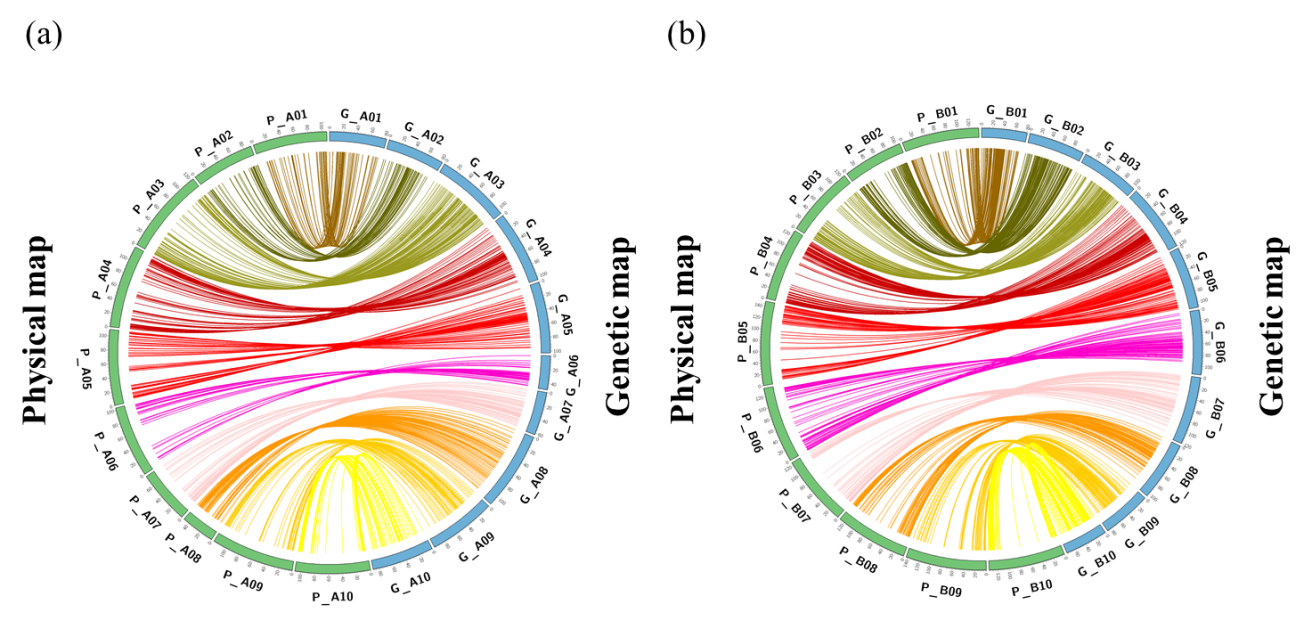


**Figure S2 Comparison of the genetic position with the physical position for the loci in the genetic map in peanut.** The chromosomes prefixed by the letter P indicated the physical map and the chromosomes prefixed the letter G indicated the genetic map in the RILs.


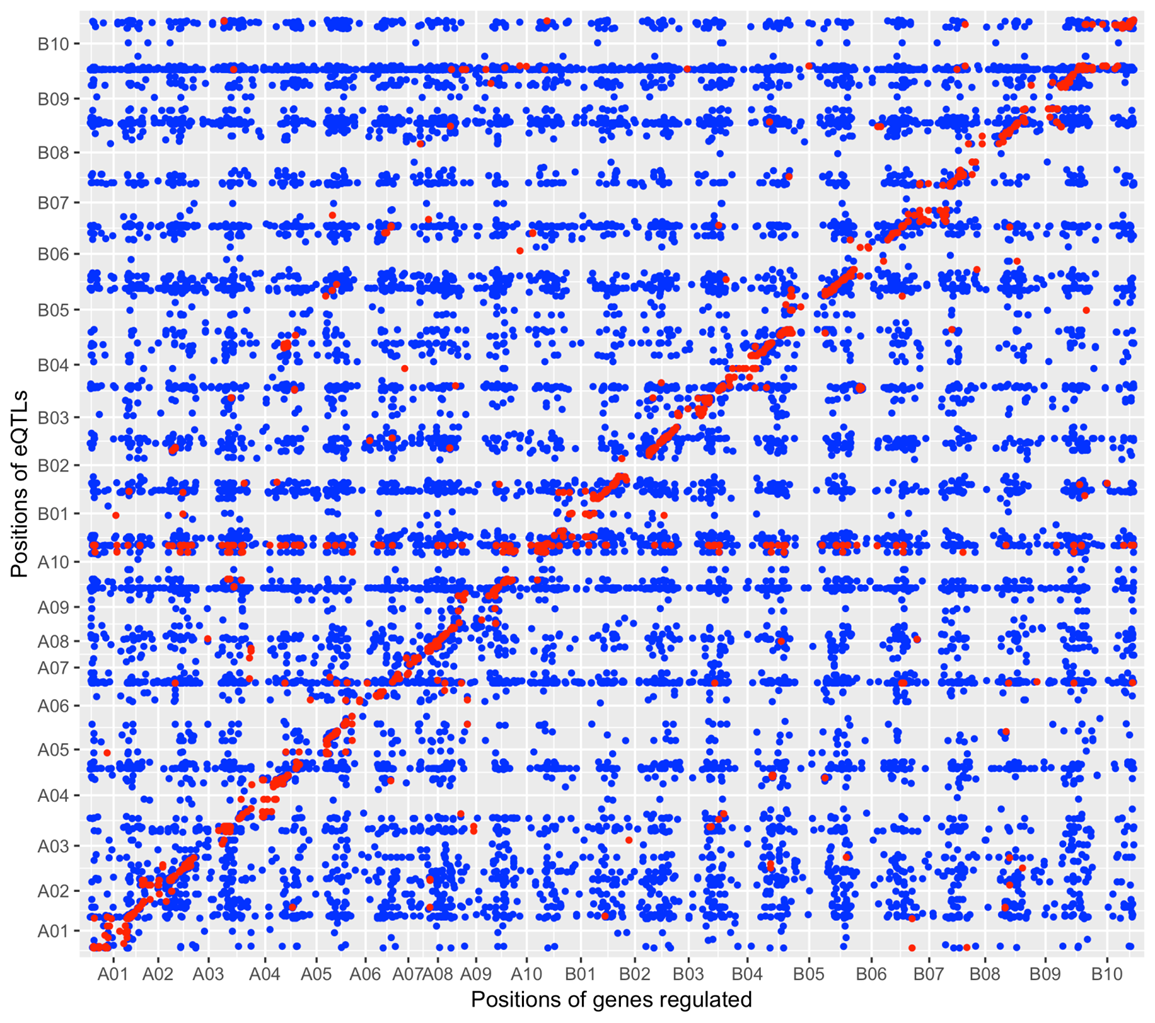


**Figure S3 The overview of eQTLs responsible for whole-genome gene expression.** The x-axis shows the genomic positions of expressed genes. The y-axis indicated the genomic positions of eQTLs. Each dot represents an eQTL for a gene expression. The dots in the diagonal indicate the local eQTLs, while those off diagonals indicate distant eQTLs. The eQTLs with *R*^2^ values greater than 20% were plotted in red, otherwise in blue.


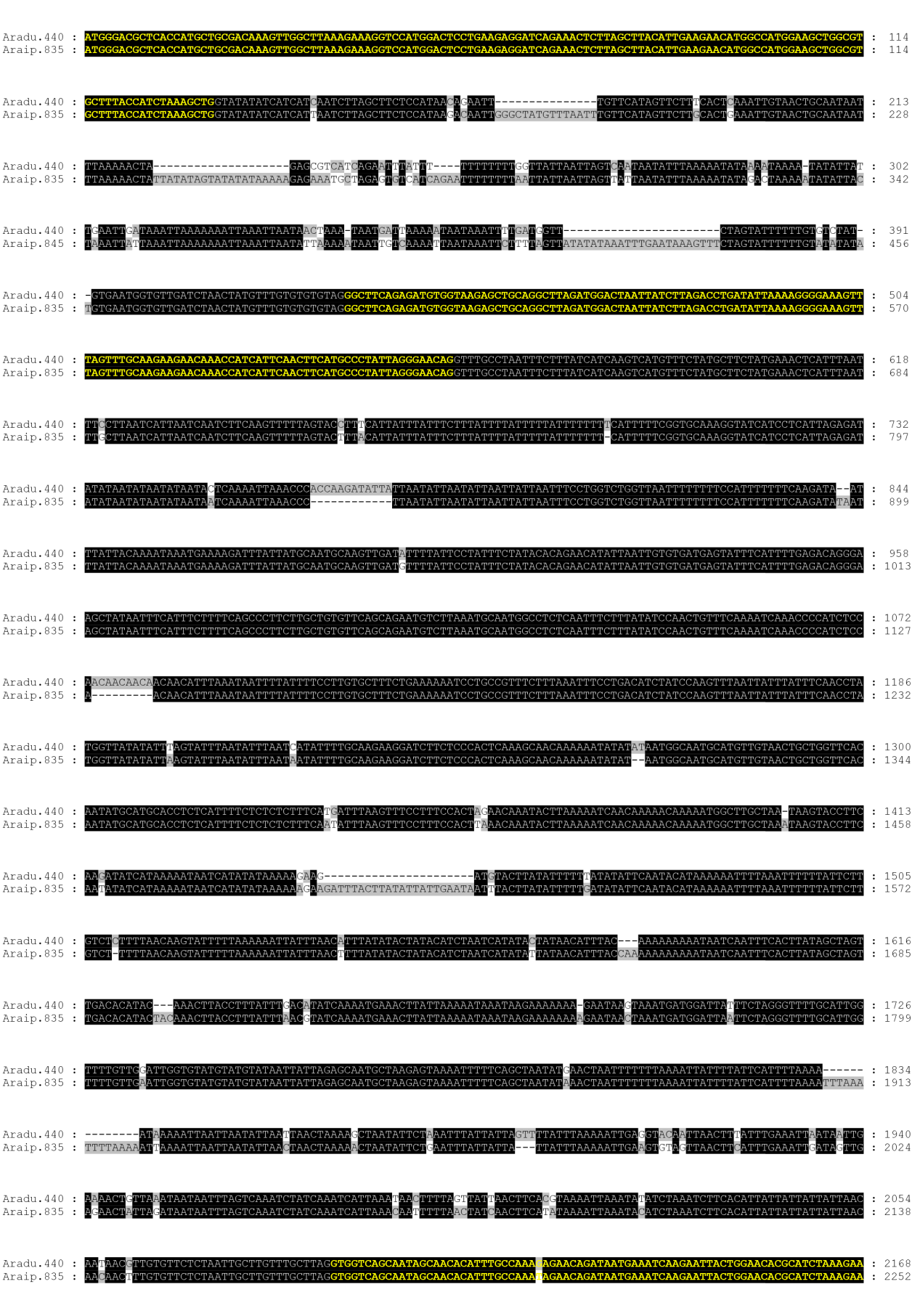

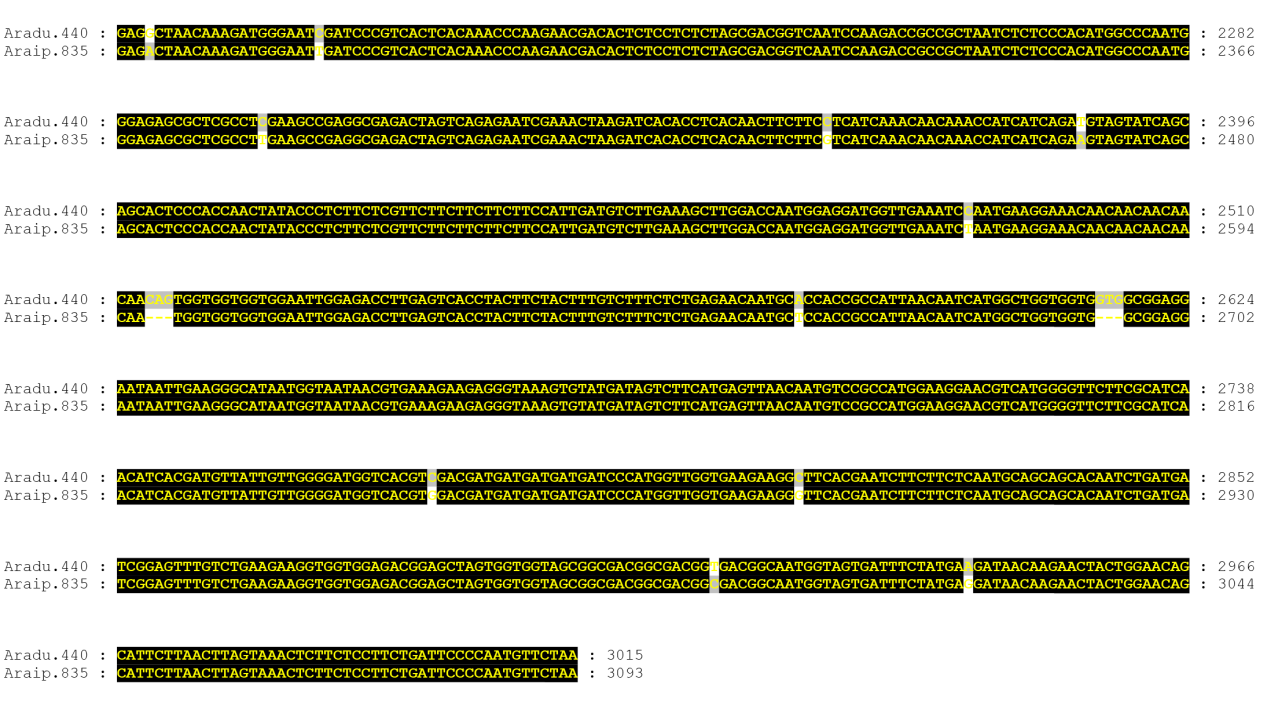


**Figure S4** Gene sequences comparing between the candidate gene *Aradu.1025440* and its homoeologous gene *Araip.10031835.* The highlighted sequences were exon sequences and the gray sequences were variations between the two genes.
